# Sampling for computational efficiency when conducting analyses in big data

**DOI:** 10.1093/aje/kwaf268

**Published:** 2025-12-05

**Authors:** Jacqueline E Rudolph, Yiyi Zhou, Karine Yenokyan, Xiaoqiang Xu, Eryka Wentz, Keri L Calkins, Corinne E Joshu, Bryan Lau

**Affiliations:** Department of Epidemiology, Bloomberg School of Public Health, Johns Hopkins University, Baltimore, MD, United States; Department of Epidemiology, Bloomberg School of Public Health, Johns Hopkins University, Baltimore, MD, United States; Department of Epidemiology, Bloomberg School of Public Health, Johns Hopkins University, Baltimore, MD, United States; Department of Medicine, School of Medicine, Johns Hopkins University, Baltimore, MD, United States; Department of Epidemiology, Bloomberg School of Public Health, Johns Hopkins University, Baltimore, MD, United States; Mathematica, Ann Arbor, MI, United States; Department of Epidemiology, Bloomberg School of Public Health, Johns Hopkins University, Baltimore, MD, United States; Department of Epidemiology, Bloomberg School of Public Health, Johns Hopkins University, Baltimore, MD, United States

**Keywords:** big data, sampling methods, case-cohort, subcohort, divide-and-recombine, computational efficiency

## Abstract

A challenge to research in big data is the inherent computational intensity of analyses, particularly when using rigorous methods to address biases. We demonstrate the use of sampling methods in big data to estimate parameters using fewer resources. Our motivating question was whether lung cancer incidence differs by baseline HIV status, using a cohort of nearly 30 million Medicaid beneficiaries. We targeted three parameters (with listed estimator): incidence rate ratio (IRR, Poisson model), HR (Cox model), and risk ratio (RR, Kaplan–Meier). We controlled for confounders using inverse probability weighting. We ran analyses using the full sample and several sampling schemes: divide-and-recombine (10, 20, 50 samples), subcohort, and case-cohort. We compared point estimates, standard errors, computation time, and memory used. We observed 1113 incident lung cancer diagnoses among 180 980 beneficiaries with HIV and 33 106 diagnoses among 29 179 940 beneficiaries without HIV. Findings were similar across target parameters. The subcohort and case-cohort approaches had estimates closer to the full sample and were faster and less memory intensive than divide-and-recombine, especially when estimating the risk ratio. Including nonsampled cases in the case-cohort resulted in increases in computation time and memory relative to the subcohort approach.

## Introduction

Big data are increasingly available in many fields of research, including epidemiology. Common sources of big data encountered in epidemiology include genome-wide association studies, insurance claims like MarketScan and Medicare, and web-scraping of search engines and social media platforms. There are no strict rules on what data are considered “big”, although the term is usually applied to datasets that either contain a large number of observations or a large number of variables (or both) and that are intensive to manage in standard software.^1^ In this paper, we focus on data with many observations.

Analyses using big data face a number of challenges, such as inconsistent data quality and missing data, but an important practical challenge to carrying out epidemiologic analyses in big data is their computational intensity. When conducting analyses in big data, epidemiologists may have to consider computation time and computer resources in a way they previously never had. One might need to benchmark their code to determine the bottleneck steps that are slowing down a program, assess which analytic approach will take less time to run, attempt to optimize code, or adapt a program to be run in parallel (ie, when multiple processes, such as bootstrap iterations, are run simultaneously).

In some cases, particularly when implementing complex methods or analyzing longitudinal data, one may still be confronted with a big-data analysis that seemingly cannot be completed in a feasible amount of time. The point at which this occurs will largely depend on 2 factors: (1) the size of the dataset and (2) the computational resources at one’s disposal (eg, using a standard laptop vs high-performing computing cluster). When faced with this challenge, one way forward is to adapt the analysis by relying on methods used in study design when other resources are limited. In particular, we can apply different sampling schema that should unbiasedly estimate our target parameter—using a fraction of the resources.

Here, we demonstrate several sampling options using an example analysis from our ongoing work. The research question of interest is whether incidence of lung cancer differs by HIV status, in a sample of nearly 30 million Medicaid beneficiaries.

## Motivating example

Our research team is investigating the intersection of HIV and cancer using Medicaid insurance claims data, from 14 US states across the calendar period 2001–2015 (Figure S1).^2^ Medicaid is a public insurance source for individuals in the United States who meet certain low-income or disability criteria. We selected Medicaid beneficiaries as our target population because 40% of people with HIV (PWH) in the United States are insured by Medicaid and because beneficiaries without HIV are inherently more similar to PWH in terms of socioeconomic status and access to healthcare than the general US population.^3^

Medicaid was further selected as a data source because the size of the population allows us to investigate a rare outcome (site-specific cancer) in the context of a rare exposure (HIV). For example, in this demonstration, we examined how incidence of lung cancer differed by baseline HIV status. The analysis included 29 360 920 beneficiaries, of whom 180 980 beneficiaries (0.6%) had HIV at baseline, and 53 528 462 total person-years of follow-up in this analysis. There were 1113 incident lung cancer diagnoses among beneficiaries with HIV and 33 106 among beneficiaries without HIV. The inclusion criteria and data structure for this analysis are described in Appendix S1 and Figure S1, and the characteristics of the study sample by HIV status are summarized in Table S1.

### Data analysis

Within these data, we targeted the average treatment effect of HIV on lung cancer. Since researchers might answer the target question in different ways, we repeated our sampling analysis estimating 3 parameters: the risk ratio (RR), HR, and incidence rate ratio (IRR). The estimation details for each parameter are described separately below. In all analyses, we controlled for potential baseline confounders of the HIV and lung cancer relationship using stabilized inverse probability weights (IPW).^4^ We controlled for sex (male/female), age (cubic splines with 2 knots), race/ethnicity (non-Hispanic Black, non-Hispanic White, Hispanic, or Other), state of residence, and calendar period at enrollment (2001–2005, 2006–2010, 2011–2015).

We first ran each analysis in the full data and then repeated our analyses using several sampling schemes:

(1) We used a divide-and-recombine approach, by splitting the data into 10, 20, and 50 random samples of equal size, running the model in each sample, and recombining using fixed effects inverse variance weighted meta-analysis.^5^ This approach was run with and without parallelization.^6^ When parallelizing, we split the subsample analyses across 10 cores; we also explored whether using 20 cores for the 20 subsamples and 50 cores for the 50 subsamples improved computational efficiency. As a sensitivity analysis, we repeated the selection of the subsamples for the 10- and 50-sample analyses 500 times and took the average of the point estimates from each iteration to assess whether our findings from the main analysis samples were due to Monte Carlo error.
(2) We used a subcohort approach with stratified sampling by exposure status, due to the rarity of the exposure. Specifically, we sampled all beneficiaries who had HIV and 10% and 25% of those without HIV. The exposure models used to estimate the IPW were weighted by the inverse of the sampling probability, and the final weights used in the outcome model were the product of the IPW and the inverse of the sampling probability.
(3) We used a case-cohort approach with stratified sampling by exposure, again sampling all beneficiaries with HIV and 10% and 25% of those without HIV.^7^ This approach built on the subcohort approach by additionally including lung cancer cases who were not selected for the subcohort (with a selection weight of 1). When estimating IRR, the nonsubcohort cases contributed 0.001 person-years of follow-up. When estimating HR and RR, the nonsubcohort cases late entered the analysis at 0.001 person-years before their event time. In this analysis, the baseline IPW were estimated in unique individuals (ie, only 1 record per person included). Similar to the sub-cohort analysis, the exposure models were weighted by the inverse of the sampling probability, and the outcome models were weighted by the IPW multiplied by the inverse of the sampling probability. The decision to weight the exposure models in the subcohort and case-cohort analyses was based on our observation that this brought point estimates closer to the full sample and appropriately modified the SE to be larger in the sampled data than the full sample (Table S2).

For a given estimand, we compared the point estimate of the target parameter in terms of absolute error (absolute difference between the sampling estimate and the full data estimate), SE, computation time in hours, and memory used in gigabytes (GB) across the sampling approaches. All analyses were run using R version 4.3.1 (The R Foundation, Vienna, Austria) on a single server node with 4 CPUs (Intel Xeon E7-4850v4 @ 2.10GHz), 64 cores, and 660GB of RAM. Code is available on GitHub.^8^

### Comparing the risk of lung cancer

We estimated the weighted RR comparing the risk of lung cancer 1, 3, and 5 years postbaseline by HIV status using the complement of the Kaplan–Meier survival estimator.^9^^,^^10^ We first present the findings for estimating the point estimate; estimation of the SE is addressed below. In the full data, the RRs at 1, 3, and 5 years were 1.66, 1.39, and 1.39 (Table 1). Estimating just those point estimates took 0.36 hours and 96.4 GB. The subcohort and case-cohort approaches had consistently low absolute error across follow-up (with perhaps the exception of the 10% subcohort at 5 years), while requiring considerably fewer resources than the full data analysis. The case-cohort analysis with a 25% sample subcohort took 0.23 hours and 36.0 GB to run; the 25% subcohort analysis took 0.19 hours and 29.4 GB. If we used a 10% sample of the unexposed, the resources used decreased further. Regardless of sample size, the case-cohort approach required more time than the subcohort, due to the inclusion of the additional nonsubcohort cases.

**Table 1:** Comparing sampling approaches for estimating the risk ratio and its SE at select years of follow-up. Note that computation time and memory used are identical across time points.

**Years follow-up**	**Approach**	**Log(RR**_**t**_ **)**	**Absolute error**	**SE**	**Time (h): Point est.**	**Memory (GB): Point est.**	**Time (h): SE**	**Memory (GB): SE**
1	Full sample	0.51	0.00	0.094	0.36	96.4	40.39	615.3
	Divide (10 samples; NP)	0.47	0.04	0.092	0.33	24.9	30.90	228.5
	Divide (10 samples; P)	0.47	0.04	NA	0.06	158.1	NA	NA
	Divide (20 samples; NP)	0.43	0.08	0.092	0.33	17.1	32.39	162.4
	Divide (20 samples; P)	0.43	0.08	NA	0.06	114.1	NA	NA
	Divide (50 samples; NP)	0.33	0.18	0.091	0.32	13.5	28.35	91.4
	Divide (50 samples; P)	0.33	0.18	NA	0.05	74.4	NA	NA
	Subcohort (25%)	0.52	0.01	0.095	0.19	29.4	20.20	376.5
	Subcohort (10%)	0.52	0.02	0.097	0.14	20.4	17.63	334.4
	Case-cohort (25%)	0.51	0.01	0.094	0.23	36.0	23.30	377.7
	Case-cohort (10%)	0.52	0.01	0.094	0.16	17.0	18.44	361.3

3	Full sample	0.33	0.00	0.071	0.36	96.4	40.39	615.3
	Divide (10 samples; NP)	0.30	0.03	0.066	0.33	24.9	30.90	228.5
	Divide (10 samples; P)	0.30	0.03	NA	0.06	158.1	NA	NA
	Divide (20 samples; NP)	0.29	0.04	0.065	0.33	17.1	32.39	162.4
	Divide (20 samples; P)	0.29	0.04	NA	0.06	114.1	NA	NA
	Divide (50 samples; NP)	0.22	0.11	0.067	0.32	13.5	28.35	91.4
	Divide (50 samples; P)	0.22	0.11	NA	0.05	74.4	NA	NA
	Subcohort (25%)	0.36	0.03	0.074	0.19	29.4	20.20	376.5
	Subcohort (10%)	0.40	0.07	0.076	0.14	20.4	17.63	334.4
	Case-cohort (25%)	0.35	0.02	0.072	0.23	36.0	23.30	377.7
	Case-cohort (10%)	0.37	0.03	0.073	0.16	17.0	18.44	361.3

5	Full sample	0.33	0.00	0.066	0.36	96.4	40.39	615.3
	Divide (10 samples; NP)	0.30	0.03	0.060	0.33	24.9	30.90	228.5
	Divide (10 samples; P)	0.30	0.03	NA	0.06	158.1	NA	NA
	Divide (20 samples; NP)	0.29	0.03	0.060	0.33	17.1	32.39	162.4
	Divide (20 samples; P)	0.29	0.03	NA	0.06	114.1	NA	NA
	Divide (50 samples; NP)	0.23	0.10	0.060	0.32	13.5	28.35	91.4
	Divide (50 samples; P)	0.23	0.10	NA	0.05	74.4	NA	NA
	Subcohort (25%)	0.34	0.01	0.068	0.19	29.4	20.20	376.5
	Subcohort (10%)	0.36	0.03	0.071	0.14	20.4	17.63	334.4
	Case-cohort (25%)	0.34	0.01	0.067	0.23	36.0	23.30	377.7
	Case-cohort (10%)	0.35	0.02	0.067	0.16	17.0	18.44	361.3

Abbreviations: B, bootstraps; Est., estimated; GB, gigabytes; h, hours; NA, not applicable; NP, no parallelization; P, parallelization; RR_t_, risk ratio at time *t*.

Whether the RR from the divide-and-recombine approach was similar to the RR from the full data depended on the number of samples and the time point at which we estimated the RR. The RR estimates from the analysis with 10 subsamples were similar to the full data. In contrast, the analysis with 50 subsamples differed regardless of the point in follow-up. When using 20 subsamples, error relative to the full data was high at 1 year but decreased by 5 years. Without parallelization, the divide-and-recombine analyses had similar computation time as the analysis in the full data but required far less memory (<25 GB). Parallelization of the divide-and-recombine analyses reduced computation time (to 0.05–0.06 hours) at the expense of memory required (increased to 114.1–158.1 GB). Allowing the analyses with 20 and 50 samples to run across an equal number of cores reduced computation time (to 0.04 and 0.02 hours, respectively) but also increased memory (to 149.1 and 269.5 GB, respectively). In the sensitivity analysis repeating the sampling 500 times, the average point estimates were similar to the point estimate from the main analysis when using 10 subsamples (log(RR) = 0.47, 0.31, and 0.31 at 1, 3, and 5 years of follow-up). When using 50 subsamples, the average log(RR) at 1 year was 0.28, which was further from the full data result than in the main analysis; in contrast, the average point estimates at 3 and 5 years were similar to the main analysis.

We used the nonparametric bootstrap to estimate the SE for the weighted RRs.^11^ Here, we specifically estimated the SE by taking the standard deviation of the bootstrap log(RR) point estimates. For the divide and recombine analyses, we took the bootstrap resamples after splitting the data so that we could obtain SEs within each subsample and use those SEs when recombining. For the subcohort and case-cohort analyses, we took the bootstrap resamples from the full data, so that the sampling process would be reflected in the estimated SE.

**Figure 1:**
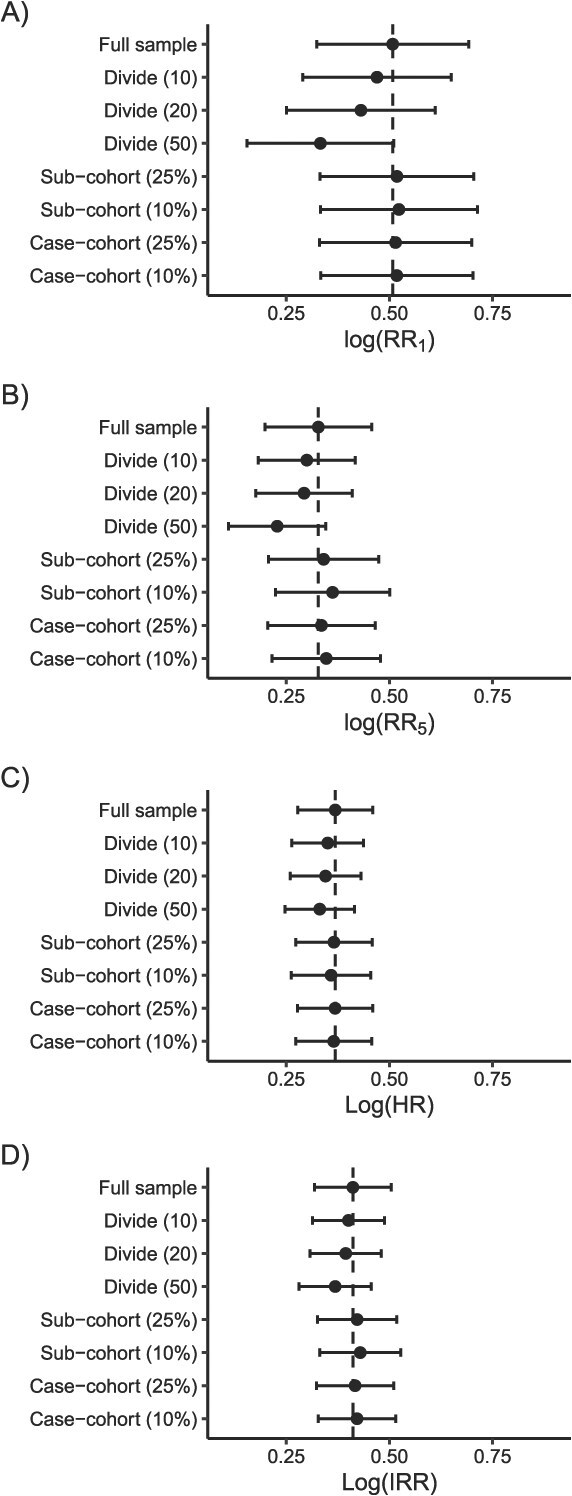
Point estimates plus 95% CIs for all approaches when estimating the (A) risk ratio (RR) at 1 year, (B) RR at 5 years, (C) HR, and (D) incidence rate ratio (IRR). For divide-and-recombine, we present the point estimate obtained from the nonparallelized analysis, and the number in parentheses refers to the number of samples. For subcohort and case-cohort, the number in parentheses refers to the size of the sample among beneficiaries without HIV.

The bootstrap is an inherently computationally intensive method, particularly when the estimation process that is repeated is already slow or has a high memory requirement. For example, based on preliminary tests showing that computation time increased linearly with number of bootstraps (Figure S2), we estimated that it would take 180 hours (7.5 days) to sequentially run the full data analysis across 500 bootstrap resamples, which is the number of resamples our study team uses as a minimum in practice. Fortunately, the bootstrap is also an easily parallelized process. As was demonstrated in the divide and recombine analysis, parallelizing greatly reduced computation time (from 0.36 to 40.39 hours for the full data). However, it also increased the memory used by the program (from 96.4 to 615.3 GB for the full data), with the memory cost increasing with the number of cores. In this analysis, we ran the bootstrap resampling across 10 cores, as that allowed us to run all 500 resamples in 1 program call within the memory constraints of the computing node. Here, we provided bootstrap SEs only for the nonparallelized divide-and-recombine analysis because parallelizing 2 nested processes is complicated. In practice, one way to accomplish the dual parallelization would be to call the analysis for each subsample in a separate, simultaneous run of the program. As illustrated in Figure 1A and B, there were minimal differences in the SE across the full data and sampling approaches. The time and memory required to run the bootstrap analyses across each sampling approach are summarized in Table 1.

### Comparing the hazard of lung cancer

We estimated the weighted HR comparing the hazard of lung cancer by HIV status using the Cox proportional hazards model. For this analysis, we estimated the SEs using the robust sandwich estimator.^12^^,^^13^ Since the variance estimation did not require the bootstrap, in our sensitivity analysis repeating the sampling 500 times, we additionally obtained the average SE. In the full data, the weighted HR was 1.45 (SE: 0.046) and took 0.43 hours and 94.7 GB to estimate (Table 2). While the divide-and-recombine approach with 50 subsamples had the largest error relative to the full data, error was low for all sampling approaches (ie, error was smaller than the SE), and SE estimates were similar across both the full data and sampling approaches (Figure 1C). We saw similar patterns in the time and memory required to run each sampling approach as was seen when estimating the RR. In the sensitivity analysis repeating the divide-and-recombine sampling 500 times, the average point estimates (10 samples: log(HR) = 0.36; 50 samples: log(HR) = 0.34) and average SEs (10 samples: 0.045; 50 samples: 0.042) were nearly identical to those found in the main analysis.

**Table 2:** Comparing sampling approaches for estimating the HR.

**Approach**	**Log(HR)**	**Absolute error**	**SE**	**Time (h)**	**Memory (GB)**
Full sample	0.37	0.00	0.046	0.43	94.7
Divide (10 samples; NP)	0.35	0.02	0.044	0.40	22.9
Divide (10 samples; P, 10 cores)	0.35	0.02	0.044	0.06	150.3
Divide (20 samples; NP)	0.35	0.02	0.044	0.39	17.5
Divide (20 samples; P, 10 cores)	0.35	0.02	0.044	0.06	108.9
Divide (20 samples; P, 20 cores)	0.35	0.02	0.044	0.04	157.8
Divide (50 samples; NP)	0.33	0.04	0.043	0.39	13.1
Divide (50 samples; P, 10 cores)	0.33	0.04	0.043	0.07	82.7
Divide (50 samples; P, 50 cores)	0.33	0.04	0.043	0.02	318.7
Subcohort (25%)	0.37	0.00	0.047	0.12	36.9
Subcohort (10%)	0.36	0.01	0.049	0.06	18
Case-cohort (25%)	0.37	0.00	0.046	0.13	37.7
Case-cohort (10%)	0.36	0.00	0.047	0.06	21.9

Abbreviations: GB, gigabytes; h, hours; NP, no parallelization; P, parallelization; RR, risk ratio.

### Comparing the rate of lung cancer

We estimated the weighted IRR comparing rate of lung cancer in beneficiaries with and without HIV using Poisson regression. As shown in Table S2, the robust sandwich SE did not appropriately capture the variability in the case-cohort analysis—the SE was smaller for the case-cohort than for the full data analysis. We thus used the nonparametric bootstrap to estimate the SE in the IRR analysis (across all sampling approaches for consistency), using the same approach described above for the RR. In the full data, the weighted IRR was 1.51 (SE: 0.048). As shown in Table 3, It took 0.41 hours and 95.3 GB to estimate the point estimate and 44.26 hours and 608.6 GB to estimate the SE across 500 bootstrap resamples. Error relative to the estimate from the full data was low across sampling approaches (again, less than the SE), and SEs were consistent (Figure 1D). Patterns in the time and memory required to estimate the point estimates and SEs across the approaches were consistent with those seen in the RR and HR analyses. In the sensitivity analysis repeating the divide-and-recombine sampling 500 times, the average point estimates (10 samples: log(IRR) = 0.40; 50 samples: log(IRR) = 0.36) were again similar to the estimates from the main analysis.

**Table 3:** Comparing sampling approaches for estimating the incidence rate ratio.

**Approach**	**Log(IRR)**	**Absolute error**	**SE**	**Time (h): Point est.**	**Memory (GB): Point est.**	**Time (h): SE**	**Memory (GB): SE**
Full sample	0.41	0.00	0.048	0.41	95.3	44.26	608.6
Divide (10 samples; NP)	0.40	0.01	0.044	0.38	27.1	34.71	231.7
Divide (10 samples; P, 10 cores)	0.40	0.01	NA	0.06	160.7	NA	NA
Divide (20 samples; NP)	0.39	0.02	0.044	0.39	17.4	37.17	130.0
Divide (20 samples; P, 10 cores)	0.39	0.02	NA	0.06	100.2	NA	NA
Divide (20 samples; P, 20 cores)	0.39	0.02	NA	0.06	150.3	NA	NA
Divide (50 samples; NP)	0.37	0.04	0.045	0.39	13.2	32.22	91.6
Divide (50 samples; P, 10 cores)	0.37	0.04	NA	0.06	78.0	NA	NA
Divide (50 samples; P, 50 cores)	0.37	0.04	NA	0.02	237.9	NA	NA
Subcohort (25%)	0.42	0.01	0.049	0.20	35.9	21.28	401.0
Subcohort (10%)	0.43	0.02	0.050	0.15	17.1	18.71	322.9
Case-cohort (25%)	0.42	0.01	0.048	0.20	36.9	22.55	391.4
Case-cohort (10%)	0.42	0.01	0.048	0.15	20.6	18.12	360.3

Abbreviations: GB, gigabytes; h, hours; NA, not applicable; NP, no parallelization; P, parallelization; RR, risk ratio.

## Discussion

Here, we demonstrated the use of sampling approaches to more efficiently estimate the association between HIV and lung cancer incidence, in a sample of almost 30 million Medicaid beneficiaries. Some of these sampling schemes, such as the case-cohort approach, are part of the standard epidemiological toolkit for study design. Here, they serve not to minimize the financial costs of designing a study but instead to minimize computation time and memory required. While the divide-and-recombine approach is more commonly found in the data science space, it has parallels to the practice of combining the results from multiple small studies using meta-analysis.

In our motivating example, which featured a rare exposure and a rare outcome, we saw that the subcohort and case-cohort approaches tended to have point estimates closer to the full sample than divide-and-recombine. The divide-and-recombine approach had higher absolute error than the other approaches, even if the 95% CIs included the full sample estimates. Additionally, divide-and-recombine only improved computation time when parallelized (at the cost of increased memory). Between the subcohort and case-cohort approach, we saw that the additional burden of including the nonsampled cases in the case-cohort analysis could result in meaningful increases in computation time and memory. One of the rationales for using the case-cohort approach over the subcohort approach is the increased statistical efficiency from including the nonsampled cases. In this analysis, though, the statistical efficiency gain was minimal. Thus, at least for our example, the subcohort approach estimated the full-data result as well as the case-cohort approach, while requiring fewer resources.

Our demonstration also showed that these sampling approaches may not always be considered necessary, depending on the target estimand and selected estimator. For example, the HR analysis was relatively fast even in the full data. That was not the case for the RR analysis, which was slow even when parallelizing the bootstrap resamples. One can easily imagine the computation time needed for a project requiring the estimation of the RR across tens of stratified and sensitivity analyses, as well as all of the iterations of analysis that occur between preliminary results and final published paper. It should be emphasized, though, that we firmly believe that one should select the target estimand based on the research question of interest, not based on whether a particular analysis will run faster. Moreover, sampling approaches could still be useful even for analyses that run in a reasonable amount of time, eg, as a way to more quickly write and debug one’s code.

The finding that the robust SE for the IRR in the case-cohort analysis was smaller than in the full data was puzzling, and we found no methodological papers in the case-cohort literature that estimated IRRs to check our results. Thus, while we were able to obtain valid SEs using bootstrap, the finding for the robust SE for the IRR deserves further thought and investigation, as this SE estimator is commonly used when obtaining weighted IRRs and when correcting for over/underdispersion. Furthermore, the robust SE worked appropriately when estimating the case-cohort HR, so this may suggest that the finding is a property of the Poisson model when using case-cohort sampling. However, further research is needed to determine (1) whether this is simply a problem in our data or a more general problem when attempting to estimate IRRs under case-cohort sampling and (2) whether the issue can be overcome, perhaps by using an alternative model for the IRR such as a Quasi-Poisson or Negative Binomial model.

We further note that we here saw lengthy computation times for what might be considered a relatively simple analysis, with just a time-fixed exposure. If one’s research question involved a time-varying exposure (and time-varying confounders), the analysis could require creating a long dataset with 1 record per time point per beneficiary. Not only will the creation of the long dataset be time-consuming; the increase in number of records could exponentially impact the resources required for estimation. Other common challenges might also impact the computation time and resources required. For example, when examining incidence in Medicaid, we usually control for informative right censoring using inverse probability of censoring weights, which similarly requires a time-varying, long dataset. We might also run a competing events analysis (with death and other cancers treated as competing events), and the Aalen–Johansen estimator of the risk function tends to have slower computation than the Kaplan–Meier estimator (at least in R). In other settings, methods for measurement error or missing data may be required, which would similarly add increased computational burden.

There are several additional points worth noting about our demonstration. First, our example featured a rare exposure and rare outcome, which could influence the observed performance of the sampling schema. This data structure is reflected in the similarity in precision between the full-sample analysis and the case-cohort and subcohort analyses. Power is driven by the smallest cell, and, despite the overall size of the data, the number of beneficiaries with HIV and lung cancer was quite low, meaning that our effective sample size was quite low. The low computational efficiency of our analysis, on the other hand, was driven by the much larger group of beneficiaries without HIV, who were on average contributing less information to estimation. While our data structure is common in epidemiological analyses of big data, as we often use big data specifically to examine rare groups, findings may differ when the effective sample size is larger (eg, if the exposure groups were more equally distributed across the 29 million beneficiaries). For instance, we expect that the divide-and-recombine approach would perform better with a more common exposure or outcome. When implementing the subcohort approach, one would likely instead choose to sample both exposure groups, which would result in lowered precision relative to the full sample.

Second, we recommend running analyses in parallel whenever feasible when analyzing big data. Setting up the parallelization will require some optimization, as one attempts to balance time with the memory used by the parallelized program. Which resource is more precious will vary by research computing setting. Third, while the slow computation time of the nonparametric bootstrap in part motivated the use of sampling approaches, we could have estimated the SE of the RR without bootstrap, if we had used a double-robust approach (eg, augmented IPW or targeted minimum loss-based estimation) with an influence function variance or a weighted parametric approach with the sandwich variance estimator.^14^^,^^15^ We could have also implemented a variant of the nonparametric bootstrap, such as the bag of little bootstraps, to improve the computational efficiency of the SE estimation.^16^

Fourth, we did not include every possible approach that could reduce computational resources. For example, if one wished to estimate the average treatment effect in the treated, a matching design could also greatly shrink total sample size, particularly if the exposure was rare, and thus require fewer resources to analyze. That is of course a different target estimand than the one estimated here, and, as mentioned above, choice of estimand should be motivated more by the research question than computation. Additionally, there are a growing number of high-performance data manipulation tools, such as duckplyr, polars, tidypolars, and data table in R.^17^ These tools are particularly useful for managing and transforming data at scale in R, as they offer notable improvements in processing speed and memory efficiency. However, their utility is limited when more complex or nuanced analyses are required—such as certain types of regression modeling or epidemiologic analyses that involve time-varying weights—that may not be fully supported within these frameworks. For this reason, these tools were not the primary focus of our paper. Nevertheless, we suggest that researchers consider combining these tools with the sampling approaches discussed here to further enhance computational efficiency when managing and analyzing large-scale epidemiologic data. Finally, we should note that the exposure-dependent sampling performed by the stratified subcohort and case-cohort approaches could induce selection bias in a measure of the overall, crude outcome incidence.^18^ Contrasts of outcome incidence or measures of incidence stratified by exposure (eg, the risk of lung cancer in PWH) would not be biased.

Epidemiologic analyses of big data are faced with both the standard challenges to inference, such as confounding, selection, or information bias, and new challenges raised by the sheer size of the analytic datasets. Here, we showed that one can apply sampling approaches to reduce the time and resources required to run an analysis in big data, while still obtaining an estimate similar to the full sample. For our motivating example, the subcohort approach was arguably the best sampling approach, particularly when estimating the RR. In big data, these sampling approaches may be all that makes feasible complex analytic methods that are time-consuming to run even in standard sample sizes.

## Supplementary Material

Web_Material_kwaf268

## Data Availability

The data that support the findings of this study are under the authority of the Centers for Medicare & Medicaid Services (CMS) and administered by ResDAC. Investigators may reuse these data if they independently meet CMS requirements and obtain both CMS reuse approval and permission from the study’s NIH program officer.
