# Correction to: Sampling for computational efficiency when conducting analyses in big data

**DOI:** 10.1093/aje/kwag152

**Published:** 2026-07-13

This is a correction to: Jacqueline E Rudolph, Yiyi Zhou, Maylin Palatino, Karine Yenokyan, Xiaoqiang Xu, Eryka Wentz, Keri L Calkins, Corinne E Joshu, Bryan Lau, Sampling for computational efficiency when conducting analyses in big data, *American Journal of Epidemiology*, Volume 195, Issue 4, April 2026, Pages 1129–1135, 10.1093/aje/kwaf268

In the originally published version of this manuscript, the third co-author Maylin Palatino was inadvertently omitted from the author list.

This error has now been corrected.

