# Supplementary material for: Sampling for computational efficiency when conducting analyses in big data
Source: Am J Epidemiol. 2025 Dec 5;195(4):1129–35. doi: 10.1093/aje/kwaf268 (PMC12862598; doi:10.1093/aje/kwaf268)
Supplement: Web_Material_kwaf268 [file web_material_kwaf268.zip › 2025-11-26_samp-big-data_supp.docx]

Sampling for computational efficiency when conducting analyses in big data

Jacqueline E. Rudolph, Yiyi Zhou, Maylin Palatino, Karine Yenokyan, Xiaoqiang Xu, Eryka Wentz, Keri L. Calkins, Corinne E. Joshu, Bryan Lau

Table of Contents

| Appendix S1. Study Sample and Data Structure | 2 |
| --- | --- |
| Figure S1. Map of the United States showing the states included in the analysis | 3 |
| Table S1. Beneficiary characteristics by baseline HIV status | 4 |
| Table S2. Comparing the model coefficients, model-based standard errors, and sandwich standard errors across different modeling decisions for the exposure models to build the inverse probability weights and the Poisson and Cox models | 5 |
| Figure S2. Increase in computation time as number of bootstrap resamples increased, when not running the bootstrap resamples in parallel | 6 |

Appendix S1. Study Sample and Data Structure

Our study sample is specifically comprised of beneficiaries who enrolled between 2001 and 2015 in 14 US states (Figure S1): Alabama, California, Colorado, Florida, Georgia, Illinois, Maryland, Massachusetts, New York, North Carolina, Ohio, Pennsylvania, Texas, and Washington. In order to ensure we have a study sample for whom we would observe all healthcare encounters, our standard inclusion criteria require beneficiaries to be between the ages of 18 and 64 years (adults who have not yet aged into Medicare), have >6 months of continual enrollment in Medicaid without dual enrollment in Medicare or private insurance, and have full Medicaid benefits (coverage is not restricted to specific care encounters, such as pregnancy-related encounters). Here, given our interest in incidence of lung cancer as first primary cancer, we additionally required beneficiaries have no cancer diagnosis prior to baseline (evidenced by the presence of one inpatient or outpatient claim with any cancer-related code).

Baseline for follow-up was defined as 6 months from the start of a beneficiary’s first eligibility period (i.e., the first period of >6 months in which a beneficiary met our inclusion criteria). Beneficiaries were then followed until diagnosis with lung cancer, diagnosis with any cancer other than lung cancer, death, disenrollment from Medicaid, enrollment in Medicare or private insurance, age 65, or administrative censoring on December 31,2015. Our exposure was HIV status at baseline, based on the presence of one inpatient claim or two outpatient claims (within two years) with an HIV-related diagnosis code. Our outcome was diagnosis with lung cancer, defined by the presence of one inpatient claim or two outpatient claims (within 2 years). Codes used to define the study sample and key variables are summarized below:

| Variable | ICD-9^a^ |
| --- | --- |
| Human Immunodeficiency Virus | 042-044, 079.53, 795.71, V08 |
| Any Cancer | 140.X-195.X, 199.X-208.X |
| Lung Cancer | 162.2-162.5, 162.8, 162.9 |

Abbreviations: ICD-9, International Classification of Diseases Ninth Edition

^a^A code ending with “.X” indicates a wildcard. Any number(s) could appear after the decimal place.


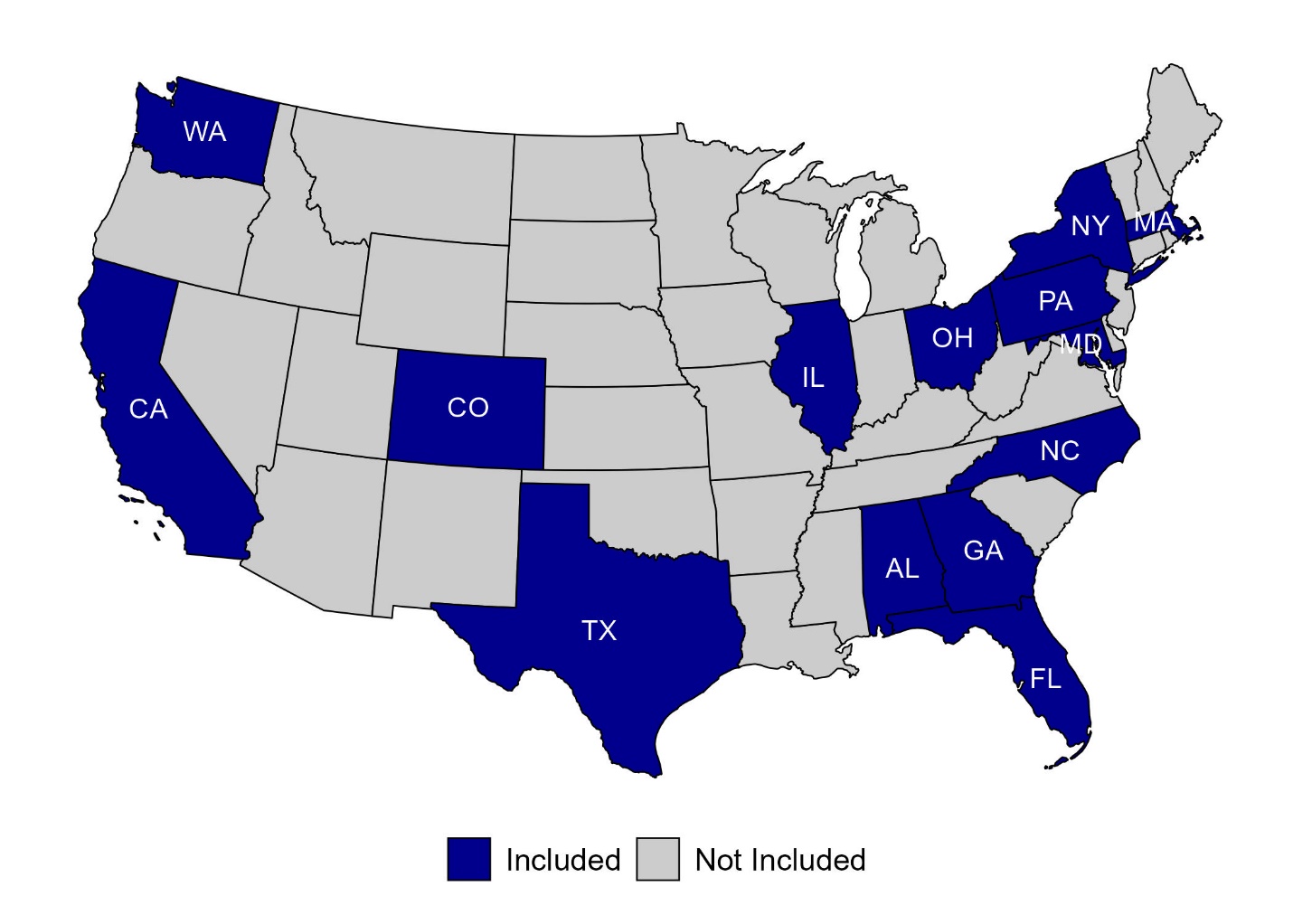


Figure S1. Map of the United States showing the states included in the analysis

Table S1. Beneficiary characteristics by baseline HIV status

| Characteristic | With HIV  (n=180,980) | Without HIV (n=29,179,940) |
| --- | --- | --- |
| Years of follow-up, median (IQR) | 1.5 (0.6, 3.7) | 0.8 (0.4, 1.9) |
| Age, median (IQR) | 42.6 (34.8, 50.1) | 28.9 (19.3, 43.9) |
| Female, n (%) | 64,577 (35.7) | 17,174,279 (58.9) |
| Race/ethnicity, n (%) |  |  |
| non-Hispanic white | 43,869 (24.2) | 11,731,599 (40.2) |
| non-Hispanic Black | 87,105 (48.1) | 6,507,853 (22.3) |
| Hispanic | 16,850 (9.3) | 5,309,039 (18.2) |
| Other/Unknown | 33,156 (18.3) | 5631449 (19.3) |
| State, n (%) |  |  |
| Alabama | 1955 (1.1) | 442,762 (1.5) |
| California | 29,668 (16.4) | 7,510,671 (25.7) |
| Colorado | 822 (0.5) | 464,728 (1.6) |
| Florida | 21,642 (12.0) | 2,167,256 (7.4) |
| Georgia | 8004 (4.4) | 1,038,867 (3.6) |
| Illinois | 9741 (5.4) | 2,415,725 (8.3) |
| Massachusetts | 7616 (4.2) | 1,324,353 (4.5) |
| Maryland | 7699 (4.3) | 815,925 (2.8) |
| North Carolina | 7053 (3.9) | 974,702 (3.3) |
| New York | 63,045 (34.8) | 4,629,718 (15.9) |
| Ohio | 5125 (2.8) | 1,947,849 (6.7) |
| Pennsylvania | 5128 (2.8) | 1,738,582 (6.0) |
| Texas | 9379 (5.2) | 2,197,918 (7.5) |
| Washington | 4103 (2.3) | 1,510,884 (5.2) |
| Enrollment period, n (%) |  |  |
| 2001-2005 | 90,009 (49.7) | 10,457,960 (35.8) |
| 2006-2010 | 33,427 (18.5) | 6,514,830 (22.3) |
| 2011-2015 | 57,544 (31.8) | 12,207,150 (41.8) |
| No. Charlson comorbidities, n (%) |  |  |
| 0 | 25,322,424 (86.8) | 120,127 (66.4) |
| 1 | 2,823,064 (9.7) | 41,650 (23.0) |
| ≥2 | 1,034,452 (3.5) | 19,203 (10.6) |

Table S2. Comparing the model coefficients, model-based standard errors (mSE), and sandwich standard errors (sSE) across different modeling decisions for the exposure models to build the inverse probability weights and the Poisson and Cox models

| Exposure  Model | Outcome  Model | Full Sample | | | Sub-cohort (25%) | | | Case-cohort (25%) | | |
| --- | --- | --- | --- | --- | --- | --- | --- | --- | --- | --- |
|  |  | Estimate | mSE | sSE | Estimate | mSE | sSE | Estimate | mSE | sSE |
|  | Poisson Model: log(IRR) | | | | | | | | | |
| Sampling weights | Combined weights | 0.411 | 0.0498 | 0.0461 | 0.408 | 0.0498 | 0.0471 | 0.411 | 0.0352 | 0.0334 |
| No weights | Combined weights | 0.411 | 0.0498 | 0.0461 | 0.415 | 0.0490 | 0.0460 | 0.425 | 0.0345 | 0.0326 |
| No weights | Sampling weights | 1.15 | 0.0305 | 0.0304 | 1.15 | 0.0305 | 0.0319 | 1.15 | 0.0215 | 0.0222 |
|  | Cox Model: log(HR) | | | | | | | | | |
| Sampling weights | Combined weights | 0.369 | 0.0498 | 0.0462 | 0.366 | 0.0498 | 0.0472 | 0.368 | 0.0352 | 0.0465 |
| No weights | Combined weights | 0.369 | 0.0498 | 0.0462 | 0.371 | 0.0490 | 0.0461 | 0.381 | 0.0345 | 0.0453 |
| No weights | Sampling weights | 1.03 | 0.0305 | 0.0306 | 1.03 | 0.0305 | 0.0323 | 1.03 | 0.0216 | 0.0311 |


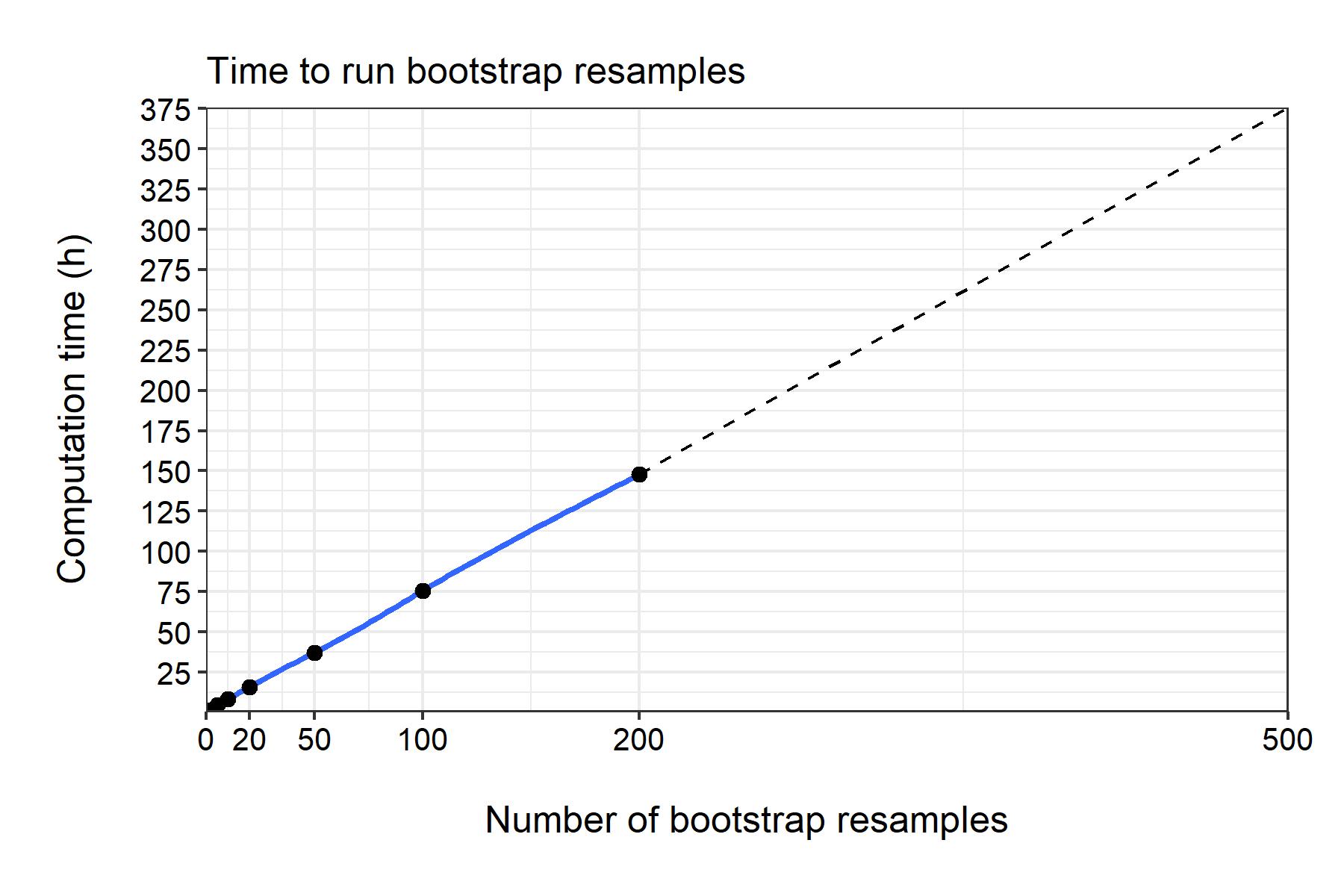


Figure S2. Increase in computation time as number of bootstrap resamples increased, when not running the bootstrap resamples in parallel
